# A comparison of two assessments of levels of functioning in clients with intellectual disability between occupational therapists and nursing staff within a long-term mental healthcare facility in South Africa

**DOI:** 10.4102/curationis.v39i1.1665

**Published:** 2016-09-27

**Authors:** Janine van der Linde, Daleen Casteleijn

**Affiliations:** 1 School of Therapeutic Sciences, University of the Witwatersrand, South Africa; 2 Department of Occupational Therapy, University of the Witwatersrand, South Africa

## Abstract

**Background:**

The implementation of the *South African Mental Health Care Act*, which regulates care for clients with intellectual disabilities, impacted on the healthcare services provided to this population. Changes in the Act necessitated planning of new care packages, which resulted in the investigation of the current hospital client profile, as well as assessment data on patient abilities according to the occupational therapist and nursing staff as primary caregivers.

**Methods:**

A retrospective, descriptive correlation study design was used as information was analysed from an existing database. Descriptive analysis of clients’ demographic data, occupational performance and adaptive functioning were done, as well as a Spearman’s rank correlation test and cluster analysis to describe the association between the levels of functioning as measured by the different professions.

**Results:**

The results indicated low levels of abilities, as well as a good to excellent correlation between results of the Fairview self-help scale and Creative Participation Assessment.

**Conclusion:**

This study provided preliminary evidence that these two tools are valuable instruments for measuring occupational performance and adaptive functioning in institutions that provide care for this vulnerable and under-researched population.

## Introduction

Witrand Hospital in Potchefstroom is one of the largest long-stay public mental healthcare institutions in South Africa, with a 650 bed-capacity for Mental Healthcare Users (MHCUs) with intellectual disabilities. The implementation of the *Mental Health Care Act* (MHCA), which regulates care for clients with intellectual disabilities, in 2005 resulted in a prolonged period of deinstitutionalisation. During this period, those MHCUs with mild to moderate abilities were integrated into the community and only clients with profound and severe disabilities were allowed to remain within the healthcare facility for care (*Mental Healthcare Act* 2002). Care plans were not adjusted accordingly and MHCUs with less ability were required to participate in activities that were inappropriate for their level of occupational performance or adaptive function. Planning of new care packages therefore required the investigation of the current hospital client profile, as well as assessment data on patient abilities, according to the occupational therapist and nursing staff as primary caregivers.

### Literature review

Within South Africa, the prevalence of intellectual disability ranges between 0.27% and 3.6% of the population compared to global numbers of 1% to 3% and the prevalence of 5% to 17% in developing countries (Kromberg *et al*. 2008; Maulik *et al*. 2011). However, limited research on intellectual disability is available in general, specifically in South Africa, and it is possible that the prevalence in the rural areas of South Africa may even be higher due to traditional beliefs where these clients are hidden because of shame (Mckenzie *et al*. 2013b). This vulnerable population receives attention in a specific section in the MHCA.

The implementation of the MHCA had a significant impact on the service delivery for people with intellectual disabilities by occupational therapists and nursing staff working in long-term public healthcare hospitals in South Africa. The Act states that only involuntary MHCUs or clients with severe and profound intellectual disabilities, as described in section seven and 26 of the Act, may receive long-term care in mental healthcare institutions as they are unable to provide consent for healthcare and are a health and safety risk for themselves as well as for other people (*Mental Healthcare Act* 2002).

Long-stay specialised institutions employ occupational therapists for their contribution within the multidisciplinary team, where they determine special needs and build on the MHCUs abilities and strengths. Occupational therapists also provide meaningful guidelines on activities and care packages for the health and wellbeing of MHCUs (Zietsman & Casteleijn 2014). They further contribute to the grouping of large numbers of MHCUs for participation in intervention programmes according to their occupational performance abilities (Zietsman & Casteleijn 2014). However, no formal guidelines are available in South Africa for the grouping of this population compared to the 21 clusters as described in the Payment by Results implemented by the NHS and in other parts of the world in terms of the clustering of clients in detailed groups (Horton 2007; Lee *et al*. 2013).

Traditionally, levels of intellectual disability were determined through IQ tests, which required a person to obtain a score approximately two standard deviations below the normal population (an IQ equal or below 70) (American Psychiatric Association 2013). This was then further categorised into degrees of impairment such as mild, moderate, severe and profound (Belva & Matson 2013). The newly published Diagnostic and Statistical Manual of Mental Disorders (DSM-5), however, states that the diagnosis of intellectual disability should rather be determined by the severity of the impact on adaptive functioning within the conceptual, social and practical domain, rather than IQ alone (American Psychiatric Association 2013). This change is welcomed by occupational therapists who rely on occupational performance in different areas and not only on intellectual functioning.

It is therefore important to view the client as a whole and ensure that all domains of function are taken into consideration as part of the multidisciplinary team evaluation. Severe and profound intellectual disability encompasses a range of difficulties such as severe cognitive disabilities, neuro-motor dysfunction, difficulties with motor and sensory functioning that requires a high level of care from nursing staff (Mckenzie *et al*. 2013b; *Mental Healthcare Act* 2002; Vlaskamp, Hiemstra & Wiersma 2007). Their added medical conditions, such as epilepsy, cerebral palsy, contractures, visual and hearing impairments and chronic respiratory difficulties, also play a role in their participation in activities (Kromberg *et al*. 2008; McKenzie *et al*. 2013a).

The contribution of nursing staff working with intellectual disabilities are thus vital in terms of the management of epilepsy and seizures, cardiac and respiratory conditions, bowel management and feeding problems (Fitzgerald & Sweeney 2013). Since nursing staff spend the most time with clients in residential care, they are best positioned to plan care pathways and to act as care-coordinators (Fitzgerald & Sweeney 2013; Hendel & Kindron 2000). Specific skills in residential care, including the management of challenging behaviour, are also mentioned by Fitzgerald and Sweeney (2013). While and Clark (2014) added competencies of nurses in intellectual disability, such as health promotion, risk assessments and communication with people involved with the individual client.

As part of routine care, a comprehensive assessment is needed to determine an individual’s occupational performance abilities and limitations. The *Mental Healthcare Act* (2002) promotes the assessment of clients with intellectual disabilities within an institution by a multidisciplinary team on a six-month to yearly basis. As the main caregivers and coordinators of care plans within this population, the occupational therapist and nursing assessment results are vital to determine the MHCUs needs and abilities (Fitzgerald & Sweeney 2013; Zietsman & Casteleijn 2014). The occupational therapist focuses on the occupational performance of MHCUs in several areas (personal management, leisure, survival skills, work and social participation), whereas nursing staff usually assess the client in terms of their medical needs and the complexity of care in terms of activities of daily living (Fitzgerald & Sweeney 2013). Assessment of the intellectually disabled population needs to be client-centred and not only determine the client’s adaptive behaviour, gender and age but also their occupational profile. A thorough analysis of their occupational performance is needed and needs to take into account the unique care establishment where the programme will be implemented (American Occupational Therapy Association 2014).

Selection of tools to assess this population can however be problematic as there are a few validated tools to use and within South Africa, there is no prescribed protocol or procedure to follow in occupational therapy or nursing assessments (Carnaby 2007; Rice 2011; Vlaskamp *et al*. 2007). Literature describes a number of tools for assessment of this specific population such as the Gunzburg progress scales (Gunzburg 1977), Fairview self-help scale (FSHS) (Ross 1970), the Matson Evaluation of Social Skills for Severely Retarded (Matson 1995b), the Vineland Adaptive Behaviour Scale (VABS) (Sparrow *et al*. 1984) and, the Developmental Assessment for individuals with Severe Disability II (DASH-II) (Matson 1995a). None of these assessments is however focused on the occupational performance of the client, being used mainly by other members of the multidisciplinary team, for example, the nursing staff.

The VABS is described in literature as the assessment most frequently used to determine adaptive behaviour from birth to adulthood in clients with intellectual disabilities and has high validity and reliability (ranged between .96 and .99) (de Bildt *et al*. 2005; Matson *et al*. 2005). The VABS is similar to the FSHS used in this study in that it determines daily self-help skills, communication skills and social skills, although motor skills and maladaptive behaviour domains are only available to specific age groups (Matson, Mayville & Laud 2003). One limitation of the FSHS is that it was developed by Ross in 1970 as a screening test and no updates were done since 1970. The advantages are that it not only covers similar domains to the popular VABS, but is also more cost effective, easier to score and shorter than the VABS and therefore easier to administer to large numbers of clients. The FSHS was found to have a good test-retest reliability between .87 and .91 over three months and a convergent validity of above .90 compared to other tests; for example, the Vineland Social Maturity scale and the Cain-Levine social competency scale (Cotten, Sison & Starr 1981).

Evidence-based assessment is needed to guide healthcare services for MHCUs with intellectual disabilities, and using an appropriate occupational therapy model is therefore useful to guide the decision-making in terms of the clients’ needs and the services that should be provided (Hawes & Houlder 2010; Lee 2010).

In the field of mental health in South Africa, occupational therapists frequently use the Vona du Toit Model of Creative Ability (VdTMoCA), developed by a local occupational therapist and suitable to the South African context (Casteleijn 2014; du Toit 1980), as a guideline for assessment and treatment (Casteleijn 2013; de Witt 2014). Having only recently been introduced to the UK, the model is steadily gaining ground as a practice model within mental health and learning disability services (Casteleijn 2014; Sherwood 2009).

According to de Witt (2014), the VdTMoCA model ‘provides a framework to assess and treat a patient’s performance in occupational performance areas of personal, interpersonal, recreational and work spheres.’ The VdTMoCA is a client-centred practice model which considers the individual’s ability to participate in occupations and the influence of the environment on participation (du Toit 1980; Kielhofner 2008). The VdTMoCA model is further based on the assumption that people’s participation in everyday activities and occupations occur according to their level of motivation and action (du Toit 1980).

Motivation and action are described as being the components of volition, which is a core concept within the creative ability theory (de Witt 2014). Motivation is described as the inner condition or drive that directs a person’s behaviour towards occupational behaviour, whereas action is seen as the conversion of motivation into a physical effort (de Witt 2014; Sherwood 2011). This is quite similar to the term volition used in the model of human occupation (MOHO), which describes the motivation to participate in occupations (Lee *et al*. 2011).

There are nine sequential levels of motivation with corresponding levels of action described within the VdTMoCA (du Toit 1980; de Witt 2014). Casteleijn (2014) conducted a study using the Rasch analysis to confirm these and found that the levels of creative ability do exist and are valid and reliable to assess a person’s level of activity participation. The first four levels, that is, tone, self-differentiation, self-presentation and passive participation, are commonly seen in MHCUs admitted to hospitals and institutions (Sherwood 2011). Three phases (therapist-directed, patient-directed and transitional phase) are described within each level to distinguish if a patient is moving from one level to another (de Witt 2014). Table 1 describes the first three levels in terms of occupational performance.

**TABLE 1:** Summary of first three levels of creative ability.

Variable	Level 1	Level 2	Level 3
Action	Unplanned action	Unconstructive action or Incidental constructive action	Constructive explorative action
Motivation/volition	Tone (similar to patients who are unconscious)	Self-differentiation	Self-presentation
	Lack awareness of themselves	Start to differentiate self from others	Want to present self to others
	Mostly unresponsive	Still very Egocentric	
Activities of daily living (self-help)	Little or no control over their bodies and bodily functions	Heavy burden of care	Constant supervision still needed for ADL’s
	24-h supervision and care	Still needs assistance and constant supervision for ADL tasks	
Work/education includes: Process skills Task concept Initiative and effort	Disorientated	Destructive actions	Explorative and willing ‘to do’
	Actions not goal directed	Unable to plan or follow instructions	Constructive 3–4 step tasks
	No task concept	No task concept, able to follow 1–2 step instruction	Partial task concept
	Non-productive in an occupational sense, no task concept	Basic concepts,	Make simple familiar activities
	Little evidence of intention or effort	Minimal initiative shown	Effort to initiate but inconsistent
Play/leisure	No awareness of play or leisure	Start to make contact with world but unintentionally, still not aware of play or leisure	Shows interest in activities and the environment, enjoy simple play/leisure activities
Social interaction/communicate	No awareness of others or different situations	Fleeting awareness of others	Awareness of basic social norms but unable to conform
	Communicate basic needs of discomfort, hunger or thirst	Express desires and refusals immediately and inappropriately	Does not initiate interaction unless for egocentric reasons
Affect/adaptive behaviour	Unable to identify extent of their distress	Behaviour unpredictable	Try to handle situations but unsure
	Bizarre behaviour may be present	Emotions limited or uncontrolled	Low self-esteem, anxiousness, behaviour impulsive

*Source*: Adapted from de Witt (2014, Table 12, 9–10)

ADL, Activities of daily living.

Little research is however available on the use of the VdTMoCA in specifically intellectual disability, but several authors indicated this is a good model to use with large groups of clients with different age groups, cultures and gender, as well as those with difficulties to judge and make their own decisions (Casteleijn & Graham 2012; de Witt 2014; Zietsman & Casteleijn 2014).

### Assessment of creative ability and adaptive functioning

Assessment within this specific learning disability population was conducted using the Creative Participation Assessment (CPA), which is based on VdTMoCA. The CPA was chosen as all occupational therapists completing the assessment were trained in the VdTMoCA and because no standardised assessments were available to measure occupational performance in terms of creative ability for this specific population.

The CPA gives a comprehensive description of the occupational performance of MHCUs and is responsive to small changes (negative or positive) that may occur over time (Casteleijn & Smit 2002; Lee *et al*. 2011). Unfortunately, there is no literature available on the use of the CPA in populations with intellectual disability, but the CPA was found to have a high construct validity and a criterion validity of .84 compared to the MOHO Volition Questionnaire and a high reliability of .70 for other MHCUs (Casteleijn 2014; Casteleijn & Smit 2002).

The CPA provides the occupational therapist with specific levels of action which guides the selection of appropriate activities for participation in pursuits in terms of ability to handle tools and materials, quality in relating to people, handling of situations and task execution, expected behaviours, norm awareness, initiative and effort and emotional responses (Casteleijn & Smit 2002).

The information gained from the CPA, as well as the information from the FSHS were used to guide the development of care packages for the population within the institution. As both the nursing staff and occupational therapists are the coordinators of care within the wards it was necessary to determine if the results of the different assessments, as done by each profession, indicate similar abilities in terms of occupational performance and the ability to participate in activities.

Translating the results of the CPA and FSHS into care plans caused some confusion in expectations of the MHCUs due to the fact the VdTMoCA gives information regarding occupational performance in different levels of motivation and action (de Witt 2014), whereas the FSHS gives information in terms of developmental level in months (Byrne & Stevens 1980; Ross 1970).

The aim of this study was therefore to collect information on the occupational performance and the adaptive functioning of the MHCUs with intellectual disabilities in order to determine the client profile within the hospital, to determine if there was a correlation between the findings of occupational therapists using creative ability and nursing staff using the FSHS, and to determine if the assessments’ findings provided information regarding clusters/groups within the institution.

## Methodology

A retrospective, descriptive correlation study design was used. The design was retrospective as an existing database, with client information and assessment results, was used and no new data were collected. Assessment data were collected from the records of MHCUs that contained the results of the CPA and FSHS.

The CPA was used to determine the MHCUs level of creative ability. This assessment uses 12 items with a seven-point scale. Each level has descriptors of the expected action and the occupational therapist then chooses the most appropriate description of the individual’s behaviour. The number of descriptors within a level are counted and the level with the highest number of descriptors, out of the 12 items, indicates the level of creative ability of the client (Casteleijn & Smit 2002).

The FSHS determines self-help skills, communication skills and social skills, motor skills and maladaptive behaviour through observation and testing. This assessment has been implemented by nurses as routine assessment and all nursing staff received training in the use of the tool. A score is generated for each domain and the final raw score is used to match with a corresponding developmental age in months between 0 and 120 months from a set chart (Fourie *et al*. 1998).

### Population and sample

MHCUs are assessed on an annual basis, as prescribed by the MHCA and hospital policy and the hospital database is updated accordingly (*Mental Healthcare Act* 2002). The records from the database contained demographic and assessment information of 663 MHCUs with intellectual disabilities in the public healthcare institution in Potchefstroom, South Africa. Only data of those MHCUs with complete demographic and assessment data, from January 2010 to December 2013 were included in the study. This resulted in a sample of 586 records. A table, developed by Kotrlik and Higgins (2001) using Cochrane’s formula, was used to determine the power calculation for the sample size and indicated that a minimum of 115 records were required. All eligible records were however included to increase the reliability and power of the data.

## Ethics and confidentiality

The study was approved by the Hospital Patient Safety Group and the North West Department of Health. All names of MHCUs and their hospital numbers were removed from the database prior to data analysis to ensure confidentiality. All the data on the database were only available to the researcher to ensure further confidentiality. The study was also approved by the Human Ethics Research Committee of the Faculty of Health Sciences from the University of the Witwatersrand (No: M130670).

### Analysis of data

Statistica Version 11 (StatSoft 2012) was used to determine the normal distribution of the sample and the correlation between the scores of the CPA and the FSHS. A cluster analysis was also performed.

The data were assessed for normal distribution using the Lilliefors analyses, which describes any value above *p* < 0.05 as not significant, therefore indicating normality. Data for age (*p* < 0.2) was not significant and therefore normally distributed, whereas data for CPA and FSHS scores, level of intellectual disability, length of stay, epilepsy and cerebral palsy were found to be significant (*p* < 0.01) .

Descriptive analysis (frequencies and percentages) of demographic data was done for age, level of intellectual disability, epilepsy and cerebral palsy. Descriptive data were also described for the average level of occupational performance on the CPA in terms of creative ability levels and average developmental functioning in months on the FSHS.

A Spearman’s rank order correlation test (Kumar 2012) was used to describe the association between the levels of functioning as measured from a nursing perspective (FSHS) and an occupational therapy perspective (CPA). A hierarchical cluster analysis, using squared Euclidean distances, was done to patterns of clusters in the data. This clustering analysis was done to indicate different naturally occurring groups; for example, to identify the number of groups and the group members’ characteristics for each measure. If the clients are the same for both measures, then seeing the correlation of the groupings could illustrate whether or not the two measures group clients in the same way.

## Results

### Demographic information

The demographic data indicated that the age of the intellectual disability population within Witrand Hospital ranged from 9 years to 95 years, with a mean age of 45 years (SD = 16) (see Table 2).

**TABLE 2:** Average chronological age of the sample (*n* = 568) for different wards.

Variable	Deinstitutionalisation preparation ward	Geriatric male	Geriatric female	Adult male with behavioural difficulty	Adult female low functioning	Protective unit: clients with mental health and behavioural difficulties	High care unit	Children’s ward
Average age per ward	66	55	46	45	42	37	40	26

*Source*: Data analysis Witrand Hospital database January 2010 to December 2013

Table 3 illustrates that the majority of clients fell within the severe intellectual disability category (*n* = 221: 39%) or the profound intellectual disability category (*n* = 165: 29%). Although the MHCA states that only profound and severely intellectual disabled clients are to be admitted, 32% of the population were only moderate (*n* = 124) or mildly (*n* = 57) disabled. It was also found that 48% (*n* = 272) of the population suffered from epilepsy, and 30% (*n* = 170) had the additional diagnosis of cerebral palsy.

**TABLE 3:** Frequency of levels of intellectual disability *n* = 568.

Intellectual Disability per ward	% Profound Intellectual Disability per ward	% Severe Intellectual Disability per ward	% Moderate Intellectual Disability per ward	% Mild Intellectual Disability per ward	Total % per specific ward
Deinstitutionalisation Preparation Ward	0	2	60	38	100
Geriatric male	24	53	19	4	100
Geriatric female	18	63	16	3	100
Adult male with behavioural difficulties	8	65	23	4	100
Adult female low functioning	27	63	10	0	100
Protective unit: clients with mental health & behavioural difficulties	0	46	34	20	100
High care unit	73	7	13	7	100
Children’s ward	86	12	0	2	100
Average for the sample	29	39	22	10	100

*Source*: Data analysis Witrand Hospital database January 2010 to December 2013

The oldest MHCUs were placed in the Deinstitutionalisation Preparation ward (average 66 years) and the youngest in the children’s ward (average 20 years). The highest percentage of profound intellectually disabled clients were found to be in the Children’s ward (86% of ward) and the High Care ward (73% of ward), whereas the most severely profound intellectually disabled clients were found in the ward with behavioural difficulties (64%), Geriatric Female ward (63%), and Female Lower Functioning ward (63%). The Deinstitutionalisation Preparation ward hosted 61% of the moderately and 39% of the mildly intellectually disabled clients.

### Functional assessment results

**FIGURE 1:**
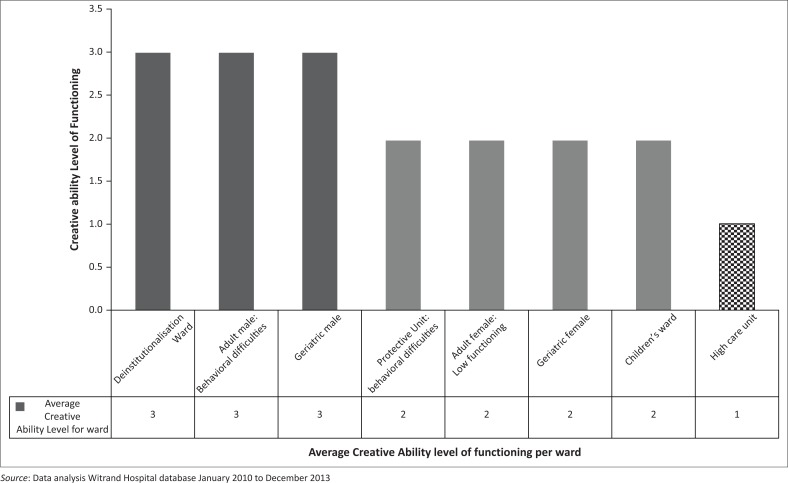
Average creative ability level of functioning per ward.

The results from the CPA indicated that MHCUs average level of occupational performance within this institution was found to be at Level 2: Unconstructive action (Figure 1). Client’s performance on this level illustrates they still need constant assistance and supervision in occupational performance tasks, have bizarre behaviours, poor social interaction and may be a danger to themselves and others.

MHCUs with the highest functioning were on Level 3 of creative ability (Level 3: Constructive explorative action). On this level, the clients are more ready to explore their environment and to participate in activities that require more steps. Their behaviour is, however, unpredictable and they require supervision. When compared with the FSHS, the MHCUs functioning ranged between 52 months for those in the deinstitutionalisation ward, 34 months for those in the ward for behavioural difficulties and 30 months for those in the Geriatric Male ward.

The MHCUs in the children’s ward, functioned on the lowest level of creative ability (Level 1: Unplanned action), six months level according to the FSHS, which indicated they needed full time care and had no concept of occupational participation.

**FIGURE 2:**
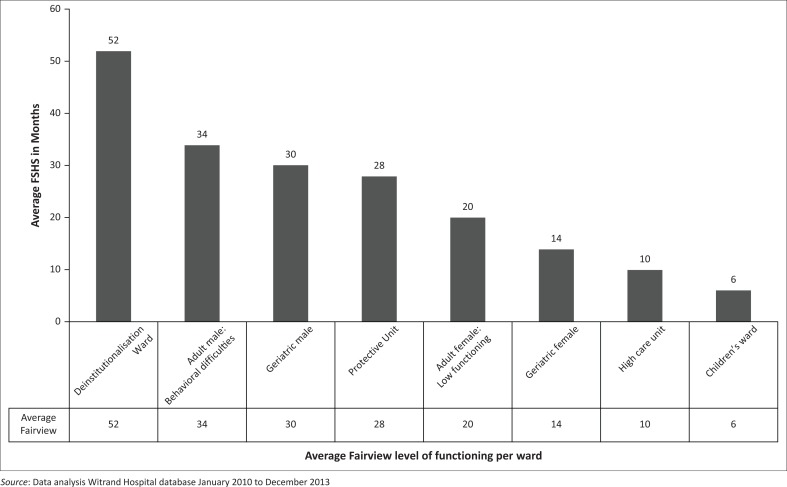
Average Fairview of functioning per ward.

The results of the FSHS assessment further indicated that the average functioning of the MHCUs is on a 24 months (two years) level. Figure 2 presents the average functioning in months per ward as measured by the FSHS.

### Correlation and cluster analysis

Spearman’s correlation was run to assess the relationship between results of the CPA and FSHS using a sample of 586 participants. Table 4 presents a strong positive correlation between levels of functioning according to the CPA and FSHS, which was statistically significant, *rs* = 0.8409, *p* = 0.0000.

**TABLE 4:** Spearman’s rank order correlations between creative ability and adaptive functioning.

Pair of variables	Spearman’s rank order correlations (Witrand patient stats) MD pairwise deleted Marked correlations are significant at *p* < 0.05000			
	Valid N	Spearman R	t(N-2)	*p*-value
Creative ability levels vs average Fairview levels	586	0.840909	37.55058	0.00

*Source*: Data analysis Witrand Hospital database January 2010 to December 2013 using Statsoft, I. (2012)

**FIGURE 3:**
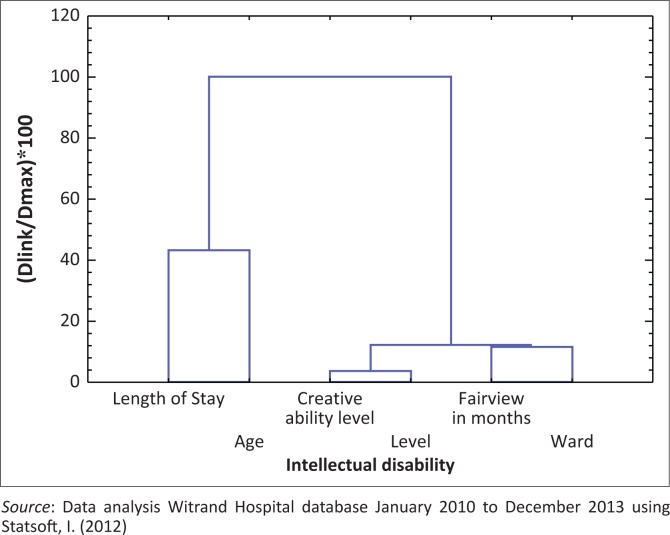
Cluster analyses Euclidean distances: Tree diagram for 6 variables.

A cluster analysis was done on the sample of 586 MHCUs with demographic data (age, length of stay, ward and level of intellectual disability) and their scores on the CPA and FSHS to determine if there were any naturally occurring groups relating to each other. A hierarchical cluster analysis, using squared Euclidean distances, indicated three clusters/groups as in Figure 3. The first cluster consisted of the level of creative ability (CPA) and intellectual disability, which indicated there was a correlation between the two variables. The second cluster consisted of the adaptive functioning (FSHS) and the wards to which the clients were admitted. A third cluster was a combination of creative ability, the level of intellectual disability, the adaptive functioning (FSHS) and the wards of the MHCUs. This third cluster illustrated that the MHCU’s level of functioning, as measured by the CPA and FSHS, was related to their level of intellectual disability.

## Discussion and implications

Levels of occupational and adaptive functioning of MHCUs were needed to design a more appropriate client-centred care plan for a group of MHCUs with intellectual disability at Witrand Hospital. The results of this study provided the necessary information to guide the healthcare team to design and implement appropriate intervention.

The average age of the MHCUs within the institution was 45 years, with a large range between 11 and 95 years of age. Petry, Maes and Vlaskamp (2007) describe age as a factor, which needs to be taken into account when choosing activities. Using MHCUs chronological age in this population may result in overestimation of their occupational performance abilities, especially when considering the literature by Ware (2003) which indicates that persons with severe and profound disabilities function on a developmental level below 2 years. Although an MHCU may physically look like a 45 year old, his or her interaction or behaviour may be slower, less consistent than that of a typically functioning person of the same age and, in some instances, even consistent with that of an infant (Ware 2003). This may not only cause an increase in stress for caregivers but may also influence the interaction and behaviour between MHCUs and staff, the choice of activities and the end products staff expect from MHCUs (de Witt 2014).

Severity of intellectual disability for classification within this population was determined on admission by the psychologist through the use of IQ testing. Unfortunately, the scores were not recorded on the hospital database. Therefore, no formal IQ scores were available to compare to the levels of occupational performance. Profound and severe intellectual disabilities were found to be the most prevalent, as 68% of the sample fell into one of these two categories. Although the MHCA states that only clients with profound or severe intellectual disabilities can be cared for in an institution, it was found that 32% of the population had a mild or moderate intellectual disability (*Mental Healthcare Act* 2002). Possible reasons for the inclusion of this group of MHCUs within the institution could be the fact that some of the clients were being prepared for discharge back into the community, or that clients within the protective unit for behavioural difficulties were held according to section 26 of MHCA, which states care for MHCUs who are incapable of making informed decisions (*Mental Healthcare Act* 2002).

Physical difficulties such as epilepsy and cerebral palsy may also influence the ability to participate in occupations within this population (Mckenzie *et al*. 2013b; Vlaskamp *et al*. 2007). MHCUs with these difficulties may have increased risk of medical complications and require careful monitoring on a full-time basis. Occupational performance is also affected by their physical abilities, for example if they have cerebral palsy. The clients age and level of intellectual and physical disability have implications on the quality of care that is provided as well as the type of care plans that can be implemented. The profound and severe intellectually disabled group especially are more dependent on nursing staff for their daily needs, as well as the type of activities they can participate in. In terms of occupational performance, Belva and Matson (2013) found this population require more assistance with activities of daily living and need to be fed, bathed and clothed, therefore requiring full time care.

The FSHS provided information regarding the adaptive behaviours of the clients in terms of self-help skills, communication skills and social interaction, as well as on motor dexterity (fine and gross motor, e.g. walking) and self-direction. The results indicated that MHCUs within this institution functioned on an average developmental level of 24 months. This is in line with the findings of Ware (2003), which describes patients with severe and profound intellectual disability functioning below 2 years developmental age. This means clients have severe performance difficulties with executing activities of daily living, communicating their needs or interacting with their caregivers or other clients.

Results of the CPA indicated the average level of occupational functioning of clients within this institution is on Level 2 of creative ability, which is on unconstructive action. Occupational therapists find on this level a person is dependent on nursing staff for assistance and unable to attend to his or her own needs. The person is aware of the environment and prepared to interact with his or her own body although only for brief periods (de Witt 2014). Although the person recognises familiar people, communication is still problematic and the concept of social norms is lacking (de Witt 2014). Zietsman and Casteleijn (2014) describe these clients as functioning on an unconstructive level, which can be seen in the heavy burden of care due to their unpredictability and the fact they are destructive in their behaviour and a consistent danger to themselves and others. No research is however available on the use of the CPA in people with intellectual disability in South Africa, although the results of this study may assist in providing preliminary data.

A good to excellent correlation was found between the FSHS and CPA. In the clinical setting, this means the two assessments provide comparable information in terms of levels of occupational performance even though the results of the levels are reflected in different values. In comparing the outcome of both the FSHS and the CPA, the results are similar in terms of the participation of self-help tasks (such a bathing, feeding, using the toilet, etc.) as well as behaviour, social interaction and communication skills.

The correlation also indicated that if the FSHS developmental level changes, the creative ability level will change in the same direction.

The first cluster indicated a relationship between the level of creative ability as measured by the CPA and intellectual disability as measured by the FSHS. The results of the CPA are consistent with their level of intellectual disability. Cluster two confirmed the fact that clients within the hospital are allocated to a ward according to their level of adaptive functioning measured by the FSHS ability and the level of care they need. A third cluster was a combination of creative ability, adaptive functioning and the wards of the MHCUs and indicated all these aspects may be interrelated.

The findings further illustrated that although the MHCUs’ occupational performance, strengths and weaknesses as measured with the different assessments indicate results in different formats, the outcome in terms of their abilities are similar. This is positive, as it will guide the occupational therapist in choosing appropriate activities, but also motivates the nursing staff in the execution of care plans as the two professions are in agreement regarding the MHCUs abilities to participate.

The study further highlighted the need for a multidisciplinary team approach and that different team members provide valuable information that is vital in planning and implementing programmes for this vulnerable population.

## Limitations

Literature on the occupational functioning of clients with intellectual disability is limited and as a result, the findings from this study could not be compared. The use of retrospective data is not ideal as unfortunately, no data were available on the IQ of clients, as well as on the phase a client is functioning on within a level of creative ability. Evidence on the validity and reliability of the CPA within the intellectual disability population is not available although the assessment was found to be valid and reliable for other mental healthcare populations. Few relevant validated tools or prescribed protocol to follow for assessments are available for use within this population and especially for a South African population (Carnaby 2007; Rice 2011). The findings of this article provide valuable preliminary information on the CPA for use in this population, but it is evident that further investigations are needed to compare or determine the appropriateness of the data.

## Conclusion

Within the South African context, the needs of people with intellectual disabilities are mainly addressed by the occupational therapy and nursing staff, and it is therefore imperative for these two professions to work together. Limited validated assessment tools are available for MHCU with intellectual disabilities, especially in South Africa, but the CPA and FSHS are valuable tools to determine the MHCUs’ needs and abilities. The types of tasks for the MHCUs will then be on their specific level of creative ability, which will result in task satisfaction and overall wellbeing.

## Key findings

Preliminary evidence suggests that the CPA and FSHS are valuable instruments for measuring occupational performance and adaptive functioning.

A multidisciplinary team approach is vital in planning and implementing care programmes.
